# Enhanced Bioavailability and Anticancer Effect of Curcumin-Loaded Electrospun Nanofiber: In Vitro and In Vivo Study

**DOI:** 10.1186/s11671-015-1146-2

**Published:** 2015-11-14

**Authors:** Chuan Wang, Chao Ma, Zhenkai Wu, He Liang, Peng Yan, Jia Song, Nan Ma, Qinghua Zhao

**Affiliations:** Department of Physical Examination Center, Shanghai First People’s Hospital, School of Medicine, Shanghai Jiao Tong University, Shanghai, 200080 People’s Republic of China; Department of Spine Surgery, Xuzhou Central Hospital, Xuzhou, Jiangsu 221009 People’s Republic of China; Department of Pediatric Orthopaedics, Shanghai XinHua Hospital, School of Medicine, Shanghai Jiao Tong University, Shanghai, 200092 People’s Republic of China; Department of Orthopaedics, Shanghai First People’s Hospital, School of Medicine, Shanghai Jiao Tong University, Shanghai, 200080 People’s R. China; Department of Orthopaedics, Shanghai XinHua Hospital, School of Medicine, Shanghai Jiao Tong University, Shanghai, 200092 People’s Republic of China; Institute for Organic and Biomolecular Chemistry, Free University of Berlin, Takustr. 3, 14195 Berlin, Germany

**Keywords:** Electrospinning, Curcumin, Bioavailability, Anticancer

## Abstract

Nanofibers have attracted increasing attention in drug delivery and other biomedical applications due to their some special properties. The present study aims to prepare a fiber-based nanosolid dispersion system to enhance the bioavailability of curcumin (CUR). CUR-loaded polyvinyl pyrrolidone (CUR@PVP) nanofibers were successfully prepared via electrospinning. Scanning electron microscopy (SEM) was employed to observe the morphology of the nanofibers, and the SEM image showed that the drug-loaded nanofibers were smooth, and no CUR clusters were found on the surface of the nanofibers. The results of X-ray diffraction (XRD) demonstrated that the CUR was evenly distributed in the nanofibers in an amorphous state. Fourier transform infrared (FTIR) spectroscopy analysis indicated that intermolecular hydrogen bonding occurred between the CUR and the polymer matrix. In vitro dissolution profiles showed that CUR@PVP nanofiber could be quickly dissolved in phosphate-buffered saline (PBS) solution, while negligible dissolution was observed in pure CUR sample. Importantly, in vitro cell viability assays and in vivo animal tests revealed that the nanosolid dispersion system dramatically enhanced the bioavailability and showed effective anticancer effect of the CUR.

## Background

Curcumin (CUR), a natural occurring phenolic substance, which was widely used as a coloring agent and traditional medicine in certain Asian countries for many centuries [1]. CUR can be extracted from many different plant species, and it has been found that CUR exhibits various pharmacological activities, including anti-inflammatory, antioxidant, antiviral, antiarthritic, and anticancer activities, as reported recently [2–8]. The detailed mechanism of these functions is complex and mainly involves cell apoptosis by modulation of complex molecular targets [9–13]. Further, toxicity tests in humans and animals proved that CUR caused no obvious physiological abnormity, even at very high doses [14]. However, CUR is insoluble at an acidic pH and unstable at an alkaline pH in water, resulting in poor gastrointestinal absorption [15, 16]. After oral administration, only a minute amount of CUR appears in the blood circulation; the majority of the administered CUR was excreted in its simplest form in the feces. As a result, despite its pharmacological efficacy and safety, this low bioavailability has restricted the clinical development of CUR [17].

Several techniques, such as nanoparticle, micelle, and liposome formation, have been reported to enhance the bioavailability of CUR [18–21]. Although these novel methods are very promising, certain limitations to their applications in medical practice still exist. The preparation technology and conditions of CUR nanoparticles are relatively complex, so these particles are not suitable for extensive production. Moreover, the biocompatibility of certain polymers used in some micelles and liposomes is still unknown, and thus, these formulations could not be applied in clinical treatment. In addition, the structural stability and loading efficiency of these formulations are also considered as restrictions to their practical use.

Solid dispersion is one of the pharmaceutical methods that can improve drug dissolution and enhance the bioavailability of poorly soluble drugs [22, 23]. This method involves the dispersion of active agents in a water-soluble polymer matrix prepared by co-melting or solvent evaporation. Typically, the release rate of the compound depends on the properties of the matrix polymer. When the solid dispersion is immersed in aqueous media, the polymer dissolves, and the medicine is released as colloidal particles in suspension, contributing to the improvement of dissolution and bioavailability [24–26]. To date, many water-soluble polymers, such as polyvinyl pyrrolidone (PVP), polyvinyl alcohol (PVA), and polyethylene glycol (PEG), have been used as dispersion matrix polymers for solubilization of poorly soluble drugs and thus for evaluation of the drugs’ bioavailability.

Electrospinning has been recognized as a simple and effective method to produce extremely fine polymer fibers with diameters ranging from micrometers to nanometers. Due to their large specific surface area, these fibers have wide biomedical applications [27, 28]. Electrospun fibers were first used as drug delivery systems (DDSs) in 2002 [29] and have been developed as an effective way of providing fast-dissolving DDSs [30–32]. Compared with the original drug form, drug-loaded fibers exhibit an extremely fast release rate. Yu et al. have developed electrospun PVP fibers as the basis of a fast-dissolving DDS capable of forming solid dispersions and improved the dissolution profiles of poorly water-soluble drugs for possible oral delivery applications [30]. To the best of our knowledge, few reports have described electrospun CUR-loaded nanofibers for in vivo investigation of cancer treatment.

**Fig. 1:**
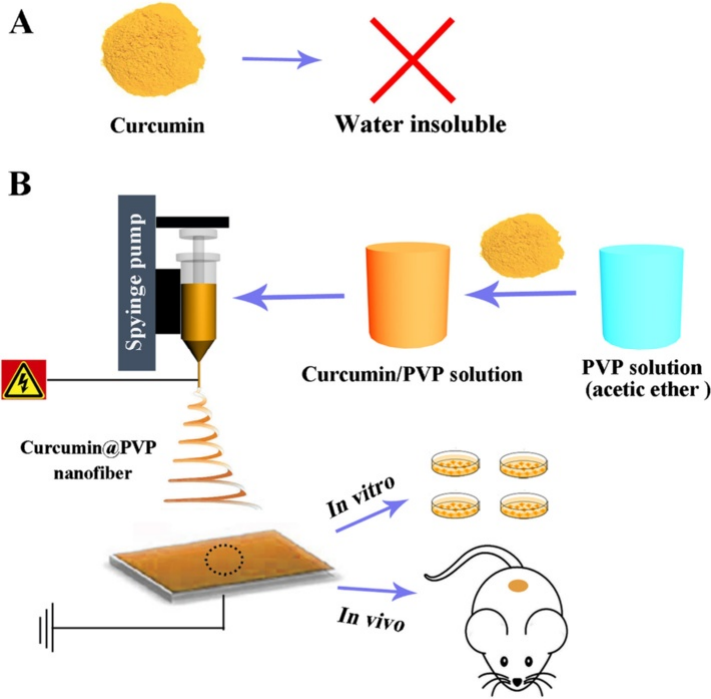
Schemtic illustration for (**a**) curicumin which are water insoluble and (**b**) the construction of CUR@PVP nanofiber and investigation of pharmacological effects in vitro and in vivo

Hence, we aimed to employ electrospun PVP nanofibers to enhance the dissolution and bioavailability of CUR, thus improving its pharmacological effects, as shown in Fig. 1. The morphology, physical, and chemical structure, in vitro drug release profiles, and anticancer effect in vitro and in vivo are investigated.

## Methods

### Materials

PVP K90 (average molecular weight = 1,300,000) was kindly donated by Chineway International Corporation (Shanghai, China). CUR powder (98 %) was purchased from Aladdin Industrial Inc. (Shanghai, China). The water used was ultra-pure, produced by a Milli-Q system (Millipore, Billerica, MA, USA). Other chemicals and reagents employed in the experiments were of analytical grade and were used as received from Sinopharm Chemical Reagent Co., Ltd. (Shanghai, China), without further purification.

### Preparation of CUR-Loaded Nanofiber Mats by Electrospinning

Spinning solutions were prepared by dissolving 1 g PVP in a 10-ml acetic ether with stirring for 4 h at 40 °C and then adding 10, 15, or 20 wt% CUR (relative to PVP) to the solution with stirring for 2 h at 37 °C. In the electrospinning process, a high-voltage power supply (BGG6-358, BMEI Co., Ltd. China) was used to provide voltage (15 kV). To avoid air bubbles, the spinning solutions were carefully loaded into a 5-ml syringe, to which a stainless-steel capillary metal-hub needle with an inside diameter of 0.5 mm was attached. The positive electrode of the high-voltage power supply was connected to the needle tip. The earthed electrode was connected to a metal collector wrapped with aluminum foil. Electrospinning was carried out under ambient conditions, and fibers were collected 15 cm from the syringe tip. The feed rate of the solutions was controlled at 2 ml/h using a single syringe pump (789100C, Cole-Parmer Instruments, USA). As-prepared samples were dried under vacuum to remove residual organic solvents before further use.

### Characterization

The morphology of the electrospun nanofibers was observed using scanning electron microscopy (SEM) (JEOL JSM-5600LV, Japan) at an acceleration voltage of 8–10 kV. Prior to imaging by SEM, samples were sputter coated with gold for 50 s to increase their conductivity. The nanofiber diameter was obtained from at least 50 measurements on a typical SEM image using Image J 1.40 G software (NIH, USA). Further examination of nanofiber fluorescence was performed on Olympus BX51 fluorescence microscope. The nanofiber samples were prepared by collecting nanofibers on a glass slide under electrospinning nozzle for 1 min and then captured using Olympus DP72 camera equipped with Olympus CellSens software. A Nicolet-Nexus 670 Fourier transform infrared (FTIR) spectrometer (Thermo Fisher Scientific, Waltham, MA) was used to obtain the FTIR spectra of the electrospun nanofibers over a range of 500–4000 cm^−1^ at a scanning resolution of 2 cm^−1^. X-ray diffraction (XRD) spectroscopy was carried out on a Rigaku D/max 2550 PC (Rigaku Inc., Japan) with Cu Kα radiation. The operating voltage and current were kept at 40 kV and 300 mA, respectively. The electrospun nanofiber samples were examined between 0 and 60° (2θ) at a scanning rate of 1° (2θ) per minute.

### In Vitro Drug Dissolution Tests

The in vitro dissolution of the CUR-loaded nanofibers and free CUR was examined in 50-ml phosphate-buffered saline (PBS; pH 7.4) at 37 °C for 48 h. In a typical process, 100 mg CUR-loaded polyvinyl pyrrolidone (CUR@PVP) nanofiber mats (containing 10 mg of CUR) and 10 mg of pure CUR powder were added into the released solvent, respectively. Subsequently, aliquots (200 μl) were withdrawn at predetermined time intervals (0.5, 1, 2, 4, 8, 12, 24, and 48 h) and then replaced with an equal volume of PBS to maintain a constant volume. At last, the samples were transformed into HPLC vials after filtering by a membrane disc filter (ME@PES Syringe Filter, Membrane Solutions, USA).

The analysis of CUR was based on previous studies [33], with modification. Briefly, the samples were performed using an Agilent 1200 high-performance liquid chromatography (HPLC) system combined with the AB SCIEX API-4000 quadrupole mass spectrometer as a detector. An Agilent Eclipse XDB-C18 (4.6 × 150 mm, 3.5 μm) was selected as the separation column and maintained at 45 °C. The mobile phase, with a total rate of 300 μl/min in gradient mode, was composed of water and methanol, each containing 0.2 % formic acid. The key parameters for CUR analysis, the declustering potential (DP), collision energy (CE), and collision cell exit potential (CXP) were fixed at −70, −50, and −12 V, respectively.

### Cell Culture

A murine melanoma cells line, denoted B16, was purchased from the Shanghai Institute of Cell Biology, Chinese Academy of Sciences (Shanghai, China). The B16 cells were maintained in RPMI 1640 medium containing 10 % FBS, 100 U/ml penicillin, and 100 μg/ml streptomycin in a humid atmosphere of 5 % CO_2_ and 95 % air at 37 °C. The medium was changed every day, and the cells were passaged by trypsinization before confluence.

### In Vitro Cytotoxicity Assessment

The cytotoxicity of the samples was evaluated using a Cell Counting Kit-8 (CCK-8; Beyotime Institute of Biotechnology, China) assay according to the manufacturer’s instructions. B16 cells were seeded at a density of 5000 cells per well on 96-well plates and allowed to settle overnight. Free CUR, free CUR dissolved with the aid of DMSO and CUR@PVP nanofiber, with various concentrations of CUR in medium (corresponding to 5, 10, 20, or 40 μg/ml CUR), were subsequently prepared and added to the 96-well plates. Cells were treated with PVP nanofibrous mats as controls. After incubation for 24 or 72 h, the cells were washed once with PBS, and 10 μl CCK-8 solution in a 90-μl serum-free RPMI 1640 medium was added to each well and then incubated at 37 °C for 4 h. The cell viability was determined by measuring the optical absorbance at 450 nm using a microplate reader (Multiskan MK3, Thermo Labsystems).

### Animal and Treatment

Naïve male C57BL/6 mice (approximately 5–7 weeks old) without specific pathogens were ordered from Shanghai SIPPR-BK Laboratory Animal Co., Ltd. Health assessment, including evaluation of the orifices, coat, and extremities, was performed on each animal every day before an experiment to avoid testing error. The room temperature was maintained at 23–26 °C with HEPA-filtered air at a rate of 15–25 air changes per hour. In addition, the relative humidity was controlled at 40–70 %, and a fluorescent light was used as illumination for a 12-h light and 12-h dark cycle. Finally, all animal experiments were humanely conducted in compliance with the Institutional Animal Care and Use Committee (IACUC) guidelines.

### In Vivo Pharmacokinetics (PK) Test

For in vivo PK testing, a total of 48 male C57BL/6 mice were randomly divided into two groups (*n* = 24 per group), and 3 mice were assigned to each pharmacokinetic time point. All tested mice needed to be fasted overnight (12 h) during the experiment to avoid the absorption of CUR being influenced by food. Free CUR or CUR@PVP nanofibrous mats were orally administered to each group at a dose of 25 mg/kg. The mice were anesthetized with 5 % isoflurane at designated time points (pre-dose and 0.25, 0.5, 1, 2, 4, 8, and 12 h) via retro-orbital injection, and blood samples (approximately 300 μl) were collected from each mouse. The collected blood, treated with the anticoagulant K_2_-EDTA, was centrifuged at 5000 rpm for 10 min at 4 °C to separate the plasma. The plasma samples were protected from light and stored at −80 °C before analysis. Prior to the analysis, plasma samples were mixed with the equal volume methanol and then centrifuged at 12,000 rpm/min in 4 °C for 10 min. The supernatant was carefully removed for LC-MS/MS analysis. The plasma drug concentration was finally calculated using external standard method with the accuracy at a 90–110 % range.

### In Vivo Anticancer Treatment

The in vivo anticancer test chiefly inspected the effect of the agents on the growth of mouse melanoma B16 cells administered to C57BL/6 mice as subcutaneous allografts. The mice were divided into three groups (each group = 20) and then treated with PVP, pure CUR, and CUR@PVP nanofibrous mats, respectively. The dosage of pure CUR and CUR@PVP nanofibrous mat groups were fixed at 50 mg curcumin per kilogram of mice body weight, and the PVP nanofiber group used as a control treated by the same amount of PVP K90.

For cancer model preparation, all mice were shaved on their right armpits and then subcutaneously subjected with a single injection of 1 × 10^6^ B16 cells in PBS. After cell injection, all groups were orally administered every day. The size of the resultant tumors was assessed blindly every day, and the tumor area (mm^2^) was assessed by measuring the maximum diameter with a caliper. At the end of the experiment, all mice were euthanatized by over-dosing pentobarbital sodium injection. The tumor of each mouse was carefully dissected from the normal subcutaneous tissue and then immersed in 4 % formalin PBS solution. After the 48-h fixation, the tumor was dehydrated with gradient alcohol-water solution and then embedded in paraffin, sectioned at 8 mm, and performed masson-trichrome (Masson) and hematoxylin & eosin (H&E) staining. At last, the stained tissue lesion was examined under a digital microscope.

### Statistical Analysis

Statistical analysis of the results was performed using one-way analysis of variance (ANOVA), followed by Student’s *t* test. The criteria for statistical significance were *P < 0.05 and **P < 0.01.

## Results and Discussion

### Morphological Analysis

**Fig. 2:**
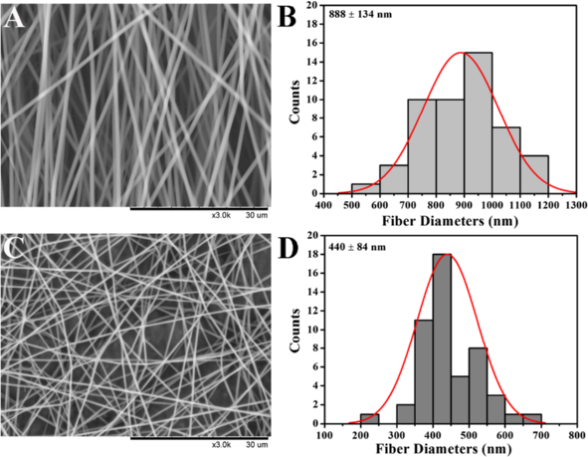
The SEM images and fiber diameter distribution histograms of (**a**, **b**) PVP and (**c**, **d**) CUR@PVP nanofiber

**Fig. 3:**
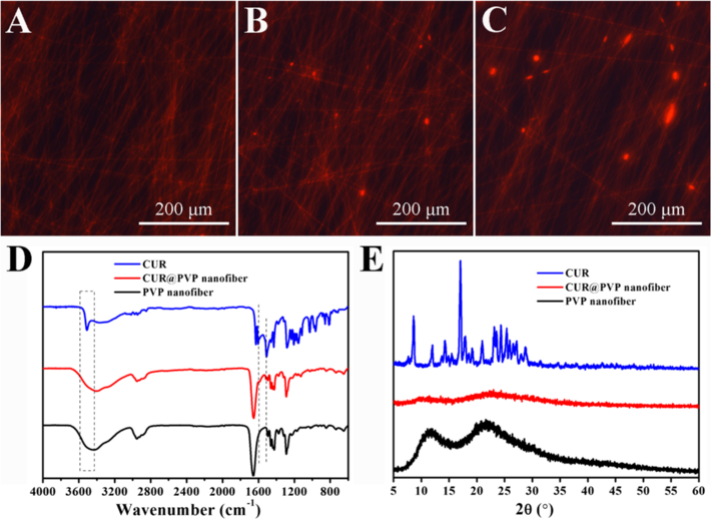
Fluorescent images of CUR@PVP nanofiber prepared with (**a**) 10 wt%, (**b**) 15 wt%, and (**c**) 20 wt% CUR. **d** FTIR spectra and (**e**) XRD data of CUR, CUR@PVP nanofiber, and PVP nanofiber

Representative SEM images of PVP and CUR@PVP nanofiber are shown in Fig. 2. It can be observed that bead-free, smooth, and uniform nanofibers were obtained. The diameter of the electrospun PVP nanofiber was 888 ± 134 nm (Fig. 2a). However, the diameter of the CUR@PVP nanofiber was sharply decreased (485 ± 123 nm) (Fig. 2b), suggesting that the incorporation of polar CUR might increase the electrospinning solution’s conductivity, thereby enhancing the electrical drawing effects on the jet fluid and decreasing the fiber fineness [34]. Fluorescence images of CUR@PVP nanofibers taken in the dark field were represented in Fig. 3a−c. It was noted that the red fluorescence of the nanofiber was observed. Because pure PVP nanofibers are not fluorescent whereas curcumin could be excited at 447 nm [35], it was reasonable to consider fibers with red fluorescence due to the uniform distribution of curcumin within PVP nanofiber matrix. We observed that when the adding amount of CUR was more than 10 wt%, CUR crystals were formed on the nanofibers. Hence, both SEM and fluorescence photos revealed that after blend electrospinning of PVP and CUR, 10 wt% of CUR was evenly incorporated within the PVP, and no crystals were separated out, indicating the electrospinning process parameters were suitable, and the method was feasible for preparation of CUR nanosolid dispersion system. Thus, CUR@PVP nanofiber prepared with 10 wt% of CUR was used in the following experiments.

### FTIR Analysis

FTIR spectral data are shown in Fig. 3d. CUR showed five characteristic peaks. The peak at 1627 cm^−1^ was due to the stretching of the C=O group, which was shifted to a lower wavenumber than usual C=O stretching (1680–1750 cm^−1^) due to conjugation present around the carbonyl group. Peaks at 1602 and 1508 cm^−1^ appeared due to aromatic C=C stretching. Phenolic C–O stretching appeared at 1427 cm^−1^, and an enolic C–O stretching peak appeared at 1281 cm^−1^. The spectrum of PVP showed a characteristic peak at 1660 cm^−1^ (C=O stretch). In addition to the significant peaks of PVP, the characteristic peaks of CUR can be observed for the CUR@PVP nanofiber, including aromatic C=C stretching at 1602 and 1508 cm^−1^, and significant broadening of peaks in the region of 3600–3400 cm^−1^ were noted, which may be attributed to intermolecular hydrogen bonding [36]. Collectively, these data indicate that either CUR or PVP retained its individual properties, and there is no chemical change due to the electrospinning approach. These findings are consistent with numerous studies which have shown that active ingredients undergo the electrospinning process without any change in their structural integrity or any loss of their effectiveness as active pharmaceutical agents [34, 37–40].

### XRD Analysis

As can be observed in Fig. 3e, the powder XRD patterns of CUR showed characteristic high-intensity diffraction peaks at 2θ values of 8.6, 11.95, 14.28, 17.08, 18, 20.87, 23.21, 24.31, and 25.41, indicating a crystalline nature. However, no characteristic diffraction peaks corresponding to CUR were observed for CUR@PVP nanofiber, suggesting of a low-order or amorphous state for the CUR.

### In Vitro Drug Dissolution Tests

**Fig. 4:**
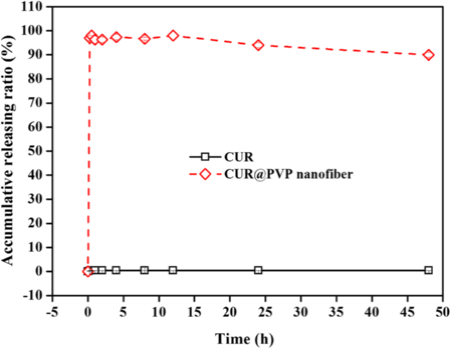
In vitro dissolution profiles of pure CUR and CUR@PVP nanofiber

Figure 4 shows the dissolution profiles of pure CUR and CUR@PVP nanofiber. As shown in Fig. 4, pure CUR showed negligible dissolution, even after 50 h. In contrast, CUR@PVP nanofiber showed a drastic increase in their dissolution rate, and the medium in the dissolution flask was yellow, indicating the presence of a stable neutral form due to the formation of a high-energy amorphous phase, as supported by the XRD data. In previous research [40], fiber mats comprising ibuprofen and PVP K30 were able to dissolve within 10 s in in vitro dissolution tests. In the present study, for CUR@PVP nanofiber, a dissolution rate above 90 % was observed within 15 min. However, the rate decreased to 89 % at 48 h. The decrease in dissolved CUR over time was predominantly caused by precipitation of the drug, which existed in a supersaturated state in buffer. The precipitated drug was visually observed after completion of the dissolution tests. These results indicate that electrospun PVP nanofibers have the potential to be used to create fast-dissolving drug release systems for poorly water-soluble drugs and their derivatives.

### In Vitro Cytotoxicity Assessment

**Fig. 5:**
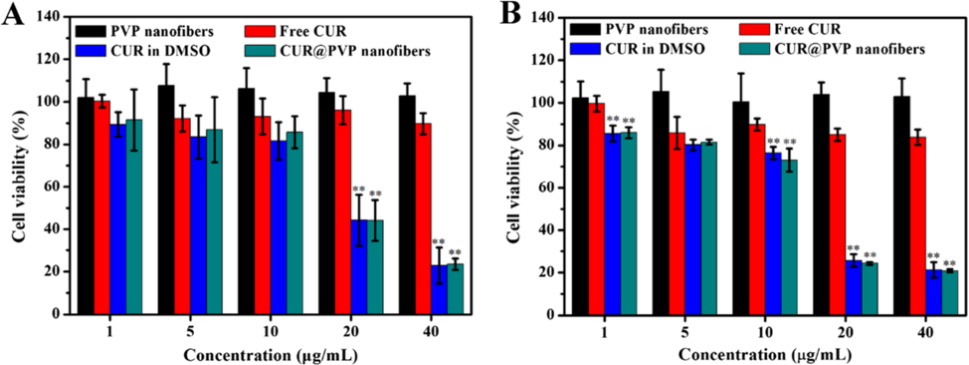
In vitro cytotoxicity of free CUR, CUR dissolved in DMSO, and CUR@PVP nanofiber was performed by testing the viability of B16 cells in CUR concentration of 5, 10, 20, and 40 μg/ml for incubation of (**a**) 24 h and (**b**) 72 h. **Significant difference compared to free CUR (*P* < 0.01)

Before conducting the in vivo experiments, the in vitro cytotoxicity of free CUR, CUR dissolved in DMSO, and CUR@PVP nanofibers was performed by testing the viability of B16 cells at CUR concentrations of 5, 10, 20, and 40 μg/ml (the relative concentrations of PVP were 50, 100, 200, and 400 μg/ml, respectively) after incubation for 24 or 72 h. The cell viability after exposure to unloaded PVP nanofibers was also investigated to eliminate the possible influence of PVP on cytotoxicity. As shown in Fig. 5, blank PVP nanofibers have nearly no obvious cytotoxicity to B16 cells at the measured concentrations within the studied time periods. We observed a drug concentration-dependent cytotoxicity to B16 cells for both CUR dissolved with the aid of DMSO (CUR in DMSO) and CUR@PVP nanofiber. As expected, both CUR in DMSO and CUR@PVP nanofiber showed significant decrease in cell viability at the CUR dose over 10 μg/ml for both 24 and 72 h, and no significant difference between them was observed. After incubation for 72 h, the cell viability for CUR@PVP was decreased to 24.4 and 20.9 % at the CUR concentrations of 20 and 40 μg/ml, respectively (Fig. 5b). Unexpectedly, in comparison, an irregular decrease in B16 cell viability was obtained for the pure CUR group. This result was probably due to the fact that a large proportion of CUR molecules were hardly soluble, and thus, the CUR was not well distributed throughout the cell medium. These results, which were consistent with the release profiles of CUR (Fig. 4), suggested that CUR@PVP nanofiber could promote the solubility of CUR, therefore exhibiting an evident growth-inhibiting effect on the B16 cells compared with free CUR.

### In Vivo PK Test

**Fig. 6:**
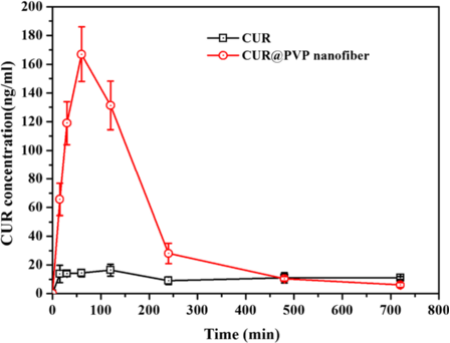
Plasma concentration vs. time profile. Plasma concentration of CUR in C57BL/6 mice. Values are mean ± SEM (*n* = 3)

Bioavailability is an important parameter, referring to the extent at which active agents are exposed to the circulation, which determines the amount of drug available to a target organ or site. Commonly, the rate of bioavailability depends on factors including the drug’s physicochemical properties (such as solubility or polarity) and formulation [41]. In the present study, CUR in mouse plasma was detected over time after oral administration of either free CUR or CUR-loaded nanofibrous mats. The PK data are summarized in Fig. 6 and Table 1. The concentrations of total curcuminoids in the plasma increased to their highest levels (167 ng/ml) at 60 min after administration and thereafter decreased gradually. Additionally, the peak concentration (*C*_max_) and the area under the curve (AUC) of the CUR-loaded nanofibrous mats were increased by 11-fold and 10-fold compared with those of free CUR, contributing to the increased bioavailability of the CUR nanosolid dispersion. This result may be attributed to the crystalline and amorphous natures of the unformulated CUR and the CUR nanosolid dispersion, respectively, as confirmed by XRD analysis. Previous studies using crystalline ritonavir, an anti-HIV drug, have demonstrated solubility-limited absorption [42], whereas the amorphous dispersion exhibited increased bioavailability because of an apparent increase in solubility.

**Table 1:** Pharmacokinetic parameters of CUR fraction following single dose oral administration of CUR and CUR@PVP nanofiber in C57BL/6 mice (*n* = 24) at 25 mg/kg

Parameter	CUR	CUR@PVP nanofiber
*C*_max_ (ng/ml)	14 ± 3	167 ± 19***
*T*_max_ (min)	120	60
AUC_0−t_ (ng.min/ml)	2720 ± 124	31,238 ± 563***

Values reported as mean ± SEM (*n* = 24). *AUC* area under the plasma concentration-time curve, *C*_*max*_ peak concentration, *T*_*max*_ time to reach peak concentration. ***P < 0.001

### In Vivo Anticancer Test

**Fig. 7:**
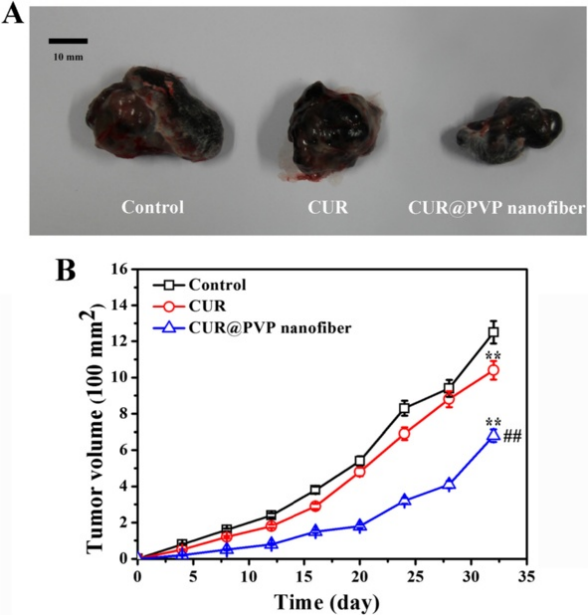
**a** Digital photo of tumor with treatment of different samples at day 32, (**b**) tumor volume vs. time profile. Values are mean ± SEM (*n* = 20). **denotes significant differences (*P* < 0.01) against control group, ## denotes significant difference (*P* < 0.01) against CUR group

**Fig. 8:**
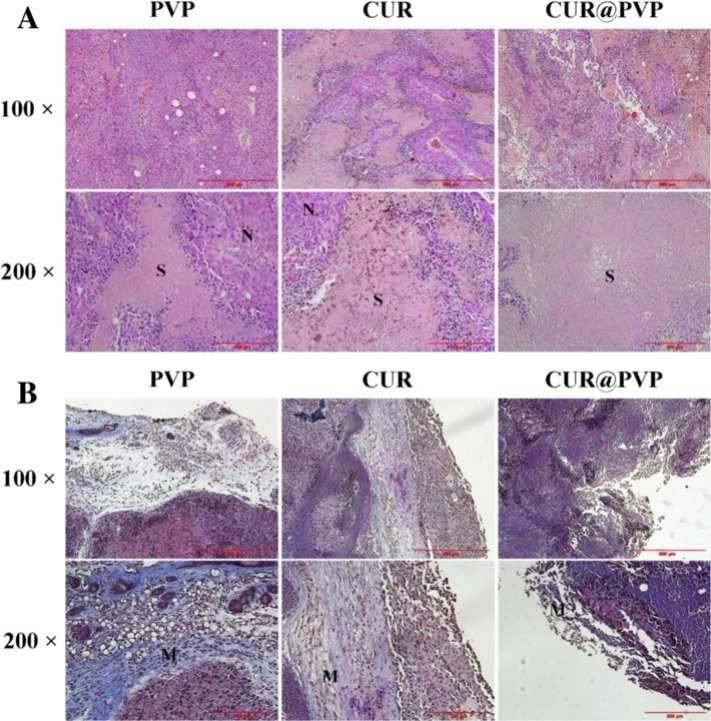
**a** H&E staining of tumors orally treated by pure PVP, CUR, and CUR@PVP nanofiber. **b** Masson staining of tumors orally treated by pure PVP, CUR, and CUR@PVP nanofiber

All animals challenged with B16 cells presented small nodules after 4 days, indicating successful model preparation. The tumor growth curve is shown in Fig. 7. It was found that the increase in the tumor size in PVP group was slightly quicker than that in the pure CUR group but visibly faster than that in the CUR@PVP group (Fig. 7a). Figure 7b revealed that the CUR@PVP group displayed much higher efficiency in inhibiting tumor growth compared with PVP group and CUR group, which was attributed to the result of the enhanced bioavailability of the CUR nanosolid dispersion, as mentioned above. The histology images of the tumor from different groups were shown in Fig. 8. H&E staining demonstrate that the allografts in mice were generated from the injected B16 cells. The tumor necrosis area was labeled as capital S, and the other tissue was recognized as N (Fig. 8a). From the micrograph, the necrosis area of CUR@PVP group is significantly larger than PVP and pure CUR groups, and pure CUR group was slightly different with PVP group, which was agreed with tumor growth curve. Masson staining revealed the tumor cell proliferation between the margin of tumor and mouse hypodermis and further illustrated tumor cell’s invasion ability (Fig. 8b). In the Masson lesion photo, the margins of each sample were labeled as M. It was demonstrated that tumor invasion ability in PVP group was most powerful, followed by pure CUR and CUR@PVP groups. From the in vivo anticancer test, we found that CUR@PVP could not only induce the cancer cell apoptosis but also fade its invasion ability.

## Conclusions

PVP nanofibers containing CUR have been successfully prepared by electrospinning. SEM analysis showed that no CUR clusters were found on the surface of the nanofibers and that the surface was smooth. Furthermore, the CUR in the composite nanofibers was presented in an amorphous state, as confirmed by XRD analysis. FTIR spectra suggested that CUR and PVP have good compatibility due to hydrogen bonding between them. In vitro dissolution tests verified that the drug-loaded PVP nanofibers were able to dissolve very quickly. Finally, the CUR@PVP nanofiber showed enhanced bioavailability and effective anticancer effect via CUR, which are promising properties for the development of CUR-based anticancer therapeutics with enhanced bioavailability.
